# Rapid replication and facile modulation of subwavelength antireflective polymer film using injection nanomolding and optical property of multilayer coatings

**DOI:** 10.1186/1556-276X-8-407

**Published:** 2013-10-02

**Authors:** Yiin-Kuen Fuh, Cheng-Chang Peng, Chieh-Tse Huang

**Affiliations:** 1 Department of Mechanical Engineering, National Central University, Jhongli 32001, Taiwan; 2 Graduate Institute of Energy Engineering, National Central University, Jhongli 32001, Taiwan; 3 Institute of Opto-mechatronics Engineering, National Central University, Jhongli 32001, Taiwan

**Keywords:** Subwavelength, Polycarbonate (PC), Injection nanomolding, Nanohole array (NHA), Reflectivity

## Abstract

A rapid, cost-effective and high-throughput process for nanotexturing subwavelength structures with high uniformity using the polycarbonate (PC) is realized via injection nanomolding. The process enables the precise control of nanohole array (NHA) surface topography (nanohole depth, diameter, and periodicity) over large areas thereby presenting a highly versatile platform for fabricating substrates with user-defined, functional performance. Specifically, the optical property of the PC substrates were systematically characterized and tuned through the modulation of the depths of NHA. The aspect ratio submicron holes can be easily modulated and experimentally proven by simply adjusting the molding temperature. The nanotextured depths were reliably fabricated in the range of 200 to 400 nm with a period of approximately 700 nm. The fabricated PC films can reduce the reflectivity from an original bare film of 10.2% and 8.9% to 1.4% and 2.1% with 400-nm depth of nanoholes at the wavelength of 400 and 550 nm, respectively. Compared with conventional moth-like nanostructures with nanopillar arrays with heights adjustable only by an etching process, this paper proposes a facile route with submicron holes to achieve a similar antireflective function, with a significantly reduced time and facile height modulation capability. Furthermore, the effects of multilayer coatings of dielectric and metallic layers on the nanomolded NHA have been performed and potential sensing application is explored.

## Background

Nanotechnology is a prioritized research topic and triggers great interest among scientists, engineers and energy researchers around the world [1,2]. Among them, surface nanotexturing has been extensively utilized in the recent years for enabling new functionalities and tailoring excellent physical and chemical properties. A wide range of examples explored recently include antireflective coatings [3,4], superhydrophobic surfaces [5,6], bio-engineered thin film [7], anti-stiction surfaces [8] and bio-mimic gecko adhesives [9]. Experimentally, artificially fabricated inverted surface patterns of NHA and high fidelity nanopillar arrays have been proposed for substrates with structural antireflective and enhanced light management properties and practical applications include high-efficiency solar cells and synthetic gecko adhesives. In particular, antireflective coating technology using thin-film stacks are mainstream tools maturely used in many optical systems [10,11]; however, critical limitations in the coating materials such as adhesion, thermal mismatch, and instability are the main drawbacks [12,13]. Therefore, nanotexturing antireflective surfaces and associated fabrication technology is booming and in great demand.

The major nanotexturing methods can be divided into the following three categories: micro-replication process (MRP) for combining micro/nanostructure masters, metallic mold electroplating, and replication into plastics [14-19]. The first primary method of MRP process can be nanoimprinting or injection nanomolding such that the mass-produce ability to functional surfaces can be implemented rapidly and is of profound technological interest [20]. The second method is roll-to-roll (R2R) manufacturing for printing organic light emitting diodes (OLED), thin-film solar cells, optical brightness enhancement films, or organic thin film transistors (OFET) [18,21-27]. The third method utilized the templates such as anodic aluminum oxide (AAO) [28,29] for anodizing high-purity aluminum to generate a porous alumina membrane as templates such that a closed-packed hexagonal array of columnar cells can be obtained. A summary for the fabrication method for the antireflective coatings is presented in Table 1.

**Table 1:** Fabrication method for the antireflective coatings

**Method**	**Characteristics**	**Applications (other than antireflective coatings)**	**References**
Micro-replication process (MRP)	Capable of creating nano/micro features on substrates of slicon or plastics. By combining three major steps of micro/nanostructure masters, metallic mold electroplating and replication into plastics.	Backlight guide plate, grating, micro-mirror arrays, photonic crystals and other micro/nano features	[14-19]
Roll-to-roll (R2R) printing	Capable of creating electronic devices on flexible substrates (plastics or metal foil) Typically includes steps of coatings, printing, laminating, re-reeling, and rewinding processes	Organic light emitting diodes (OLED), thin film solar cells, optical brightness enhancement films or organic thin film transistors (OFET)	[18,21-27]
Anodic aluminum oxide (AAO)	By anodizing high-purity aluminum to generate a porous alumina membrane as templates such that a closed-packed hexagonal array of columnar cells can be obtained. Typically, can be categorized as a self-ordering synthesis of nanopores	Molecular separation, energy generation and storage, electronics, photonics, sensors (biosensors), drug delivery, and template synthesis	[28,29]

In this paper, we present a facile and fast fabrication route for high-throughput, low-cost nanotexturing of surfaces with tunable NHA depths. The optical properties of the textured films were systematically characterized as a demonstration to validate the proposed technique for enabling substrates with functional performance of tunable reflectivities. In addition, this NHA can be integrated with multilayer coatings of both metallic and dielectric layers to further tailor the optical properties such as ultra-sensitive sensing applications.

## Methods

### Preparation of the PC film via precision injection nanomolding

**Figure 1:**
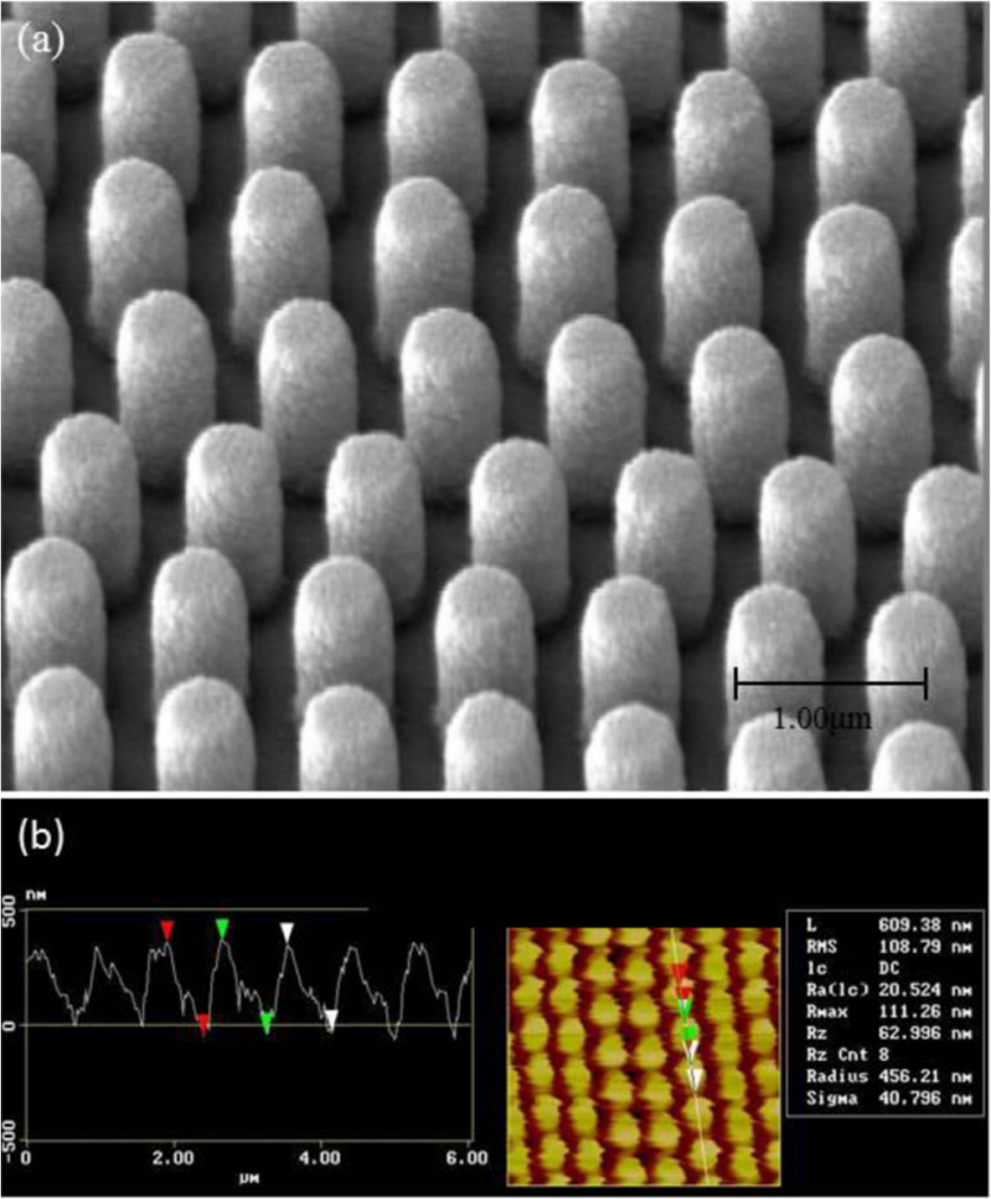
**SEM (a) and AFM images (b) of Ni stamp used for injection nanomolding experiment.** The period of the nanopillar array in the Ni stamp is about 700 nm and the depth is about 400 nm.

**Figure 2:**
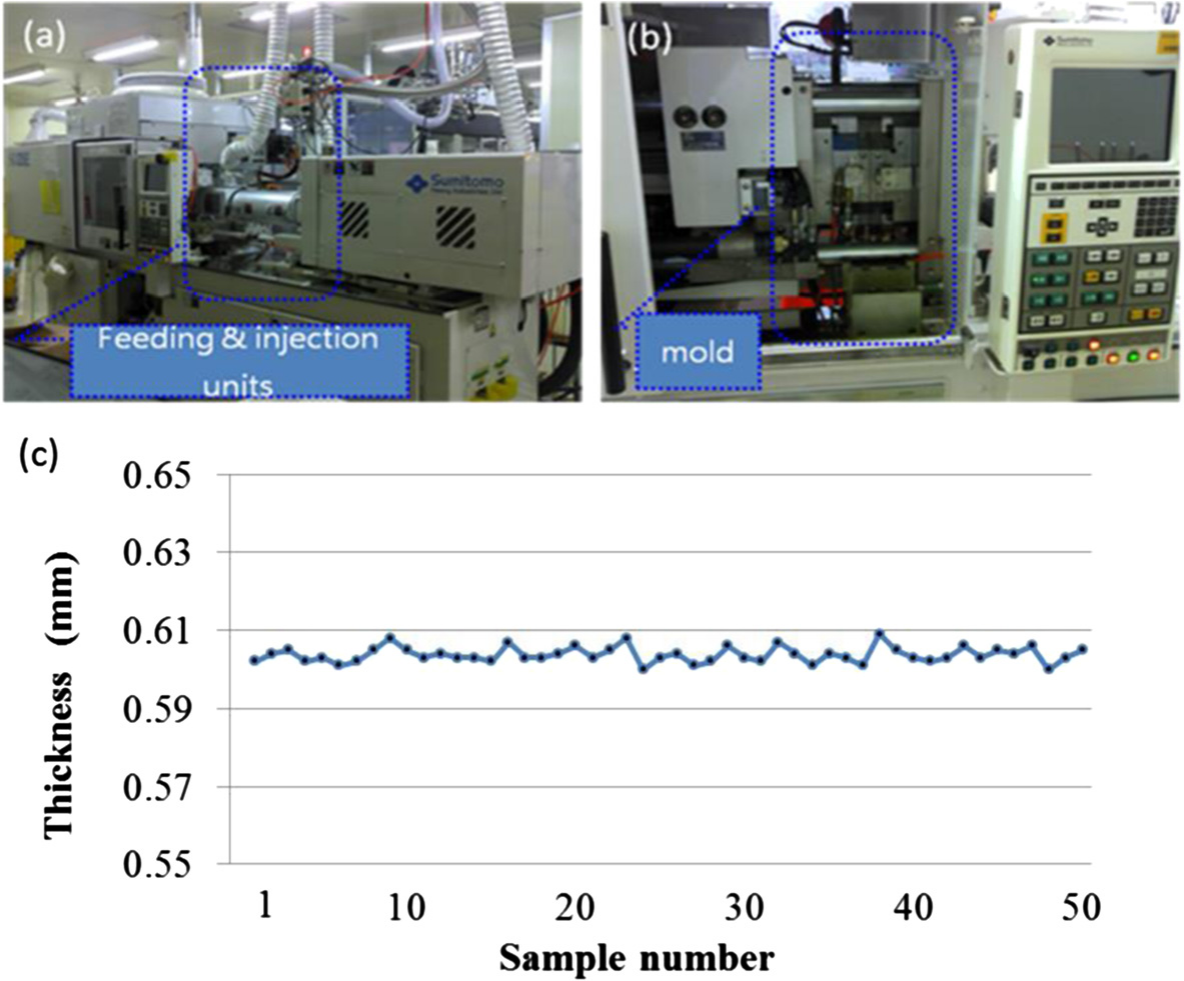
**Precision injection nanomolding equipment used for experiments and precisely replicated NHA controlled by nanoinjected substrate thickness.** Experiments showing **(a)** feeding and injection units and **(b)** mold region for the nanotextured Ni stamp. **(c)** Importance of precisely replicated NHA being carefully controlled by the nanoinjected substrate thickness.

Precision injection nanomolding processes were routinely used to fabricate optical disks in large quantities such as CD, DVD, and blue-ray disks (BD) with subwavelength features. Therefore, we chose precision injection nanomolding to fabricate the optical element with submicron holes. Due to high optical transparency in the visible and near-infrared wavelengths, polycarbonate (PC) pellets (TAIRILITE, MD1500, 99.5% pure) were chosen as the polymer materials. A critical issue of nanoimprint or nanostructure replication is the fabrication of nanostructured stamp. Previously, the nickel imprint stamp using electroforming process and features as small as 50-nm-sized patterns of original silicon master were faithfully transferred [30]. The details of the electroforming process such as composition of the chemical solution and operating parameters can be found in [31]. For the Ni mold used for the injection nanomolding, similar to the optical disk production and prior studies, electroforming is adopted to transfer the nanostructures with the original master silicon molds. Figure 1 shows both scanning electron microscope (SEM) and atomic force microscope (AFM) images of the Ni mold used. The period of the Ni mold array is in the range of 650 to 700 nm and the nanopillar heights are about 400 nm. Precision injection nanomolding machine (Sumitomo SD35E) used for the experiments were shown in Figure 2 and the feeding and injection units can be clearly seen respectively in Figure 2a. The mold region where the Ni mold resides is also indicated in Figure 2b. Furthermore, Figure 2c illustrated the importance of precisely replicated NHA being carefully controlled by the nanoinjected substrate thickness. The experimental results reveal that the standard deviations of 50 selected samples for substrate thickness can be reliably minimized to 0.02%, demonstrating the highly consistent capability in the nanoreplication process.

### Characterization of the replication process and operating parameters

To characterize the nanotextured surfaces, both SEM (LEO 1530 Gemini, Zeiss, Oberkochen, Germany) and AFM (Digital Instruments nanoscope, Tonawanda, NY, USA) were utilized. For the optical reflectivity measurements, spectrophotometer STEAG ETA-Optic (Heinsberg, Germany) and n&k analyzer 1280 (n&k Technology, Inc., San Jose, CA, USA) were used at the angle of 90°.

## Results and discussion

The precision injection nanomolding process has been widely accepted as one of the rapid replication methods to transfer nanostructures and is considered a major mass production technique for a wide range of commercial products [13]. In particular, the major processing parameters can be classified into the following: injection and mold temperatures, packing time and pressure, injection speed, etc. The diameter of the injection nanomolded film is a disk shape which geometric dimension is 120 mm in diameter and 0.6-mm thick. For a typical injection nanomolding operation, the following parameters apply: mold temperature is intentionally controlled in the range of 115 to 130°C, respectively, while the following parameters are fixed: 0.5-s packing time and 130-MPa packing pressure, injection speed 120 cm/s while the PC viscous flow was maintained at 320°C, total clamping force is fixed at 350 KN. Total cycle time for one shot of process including automatic transfer can be as low as 4 s while maintaining a high-fidelity replication. An automatic monitoring system is included in the injection process and deviation for the molding temperature is within ±0.5°C. In previous studies, the molding and PC flow temperature play a significant role on the replicated structure, both in terms of precise fidelity of depth and pitch. Other experimental work can be briefly explained as following: a stock PC pellets is fed into the system and used as the supply material. The mold holds a temperature controlled water circulation system for the purpose of heating and cooling function that facilitates the continuous operation and to ensure uniformity of viscous flow. The NHA stamp is held in the machine firmly and symmetrically about the mold geometric center while the transfer mechanism is concurrently applied. Upon finishing the molding process, the molded part is transferred to a conveyer for later rinsing deionized (DI) water bath. The system allows the user to control all the above parameter settings, and in particular, both the material and the molding temperatures are the most crucial ones.

**Figure 3:**
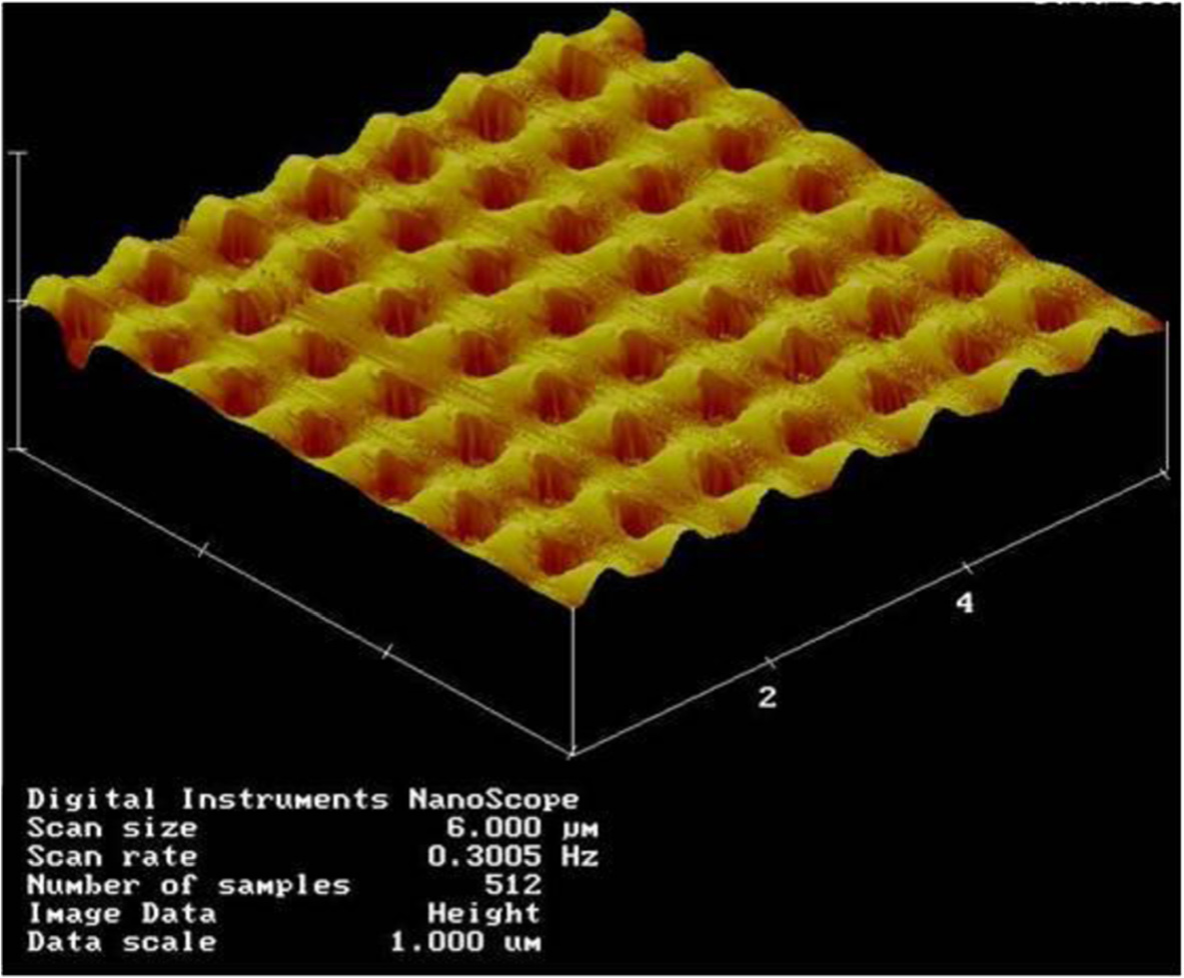
**AFM micrograph of a typical PC film with injection nanomolded submicron holes.** The scanned area is 6 μm × 6 μm.

Figure 3 shows AFM image of a typical replication of submicron holes with a scan area of 6 × 6 μm^2^. Submicron holes can be reliably and swiftly replicated for the scanned areas, and typically, we select five to seven measurements for the uniformity consideration. The fidelity of replication is experimentally validated to be extremely good and deviations are routinely maintained with 10% of the fabricated NHA depths. Previous experiences from CD/DVD/BD manufacture assist us in choosing the molding temperature as the dominating factor in the nanoreplication process. In order to investigate the impact of different molding temperatures, temperatures in the range of 110°C to 130°C are selected for the PC film replication process. From Figure 3, a uniformity of NHA can be demonstrated in cross-sectional view of a 700-nm pitch sample, showing consistently conformal NWA diameters of approximately 400 nm and 300-nm depth can be fabricated.

**Figure 4:**
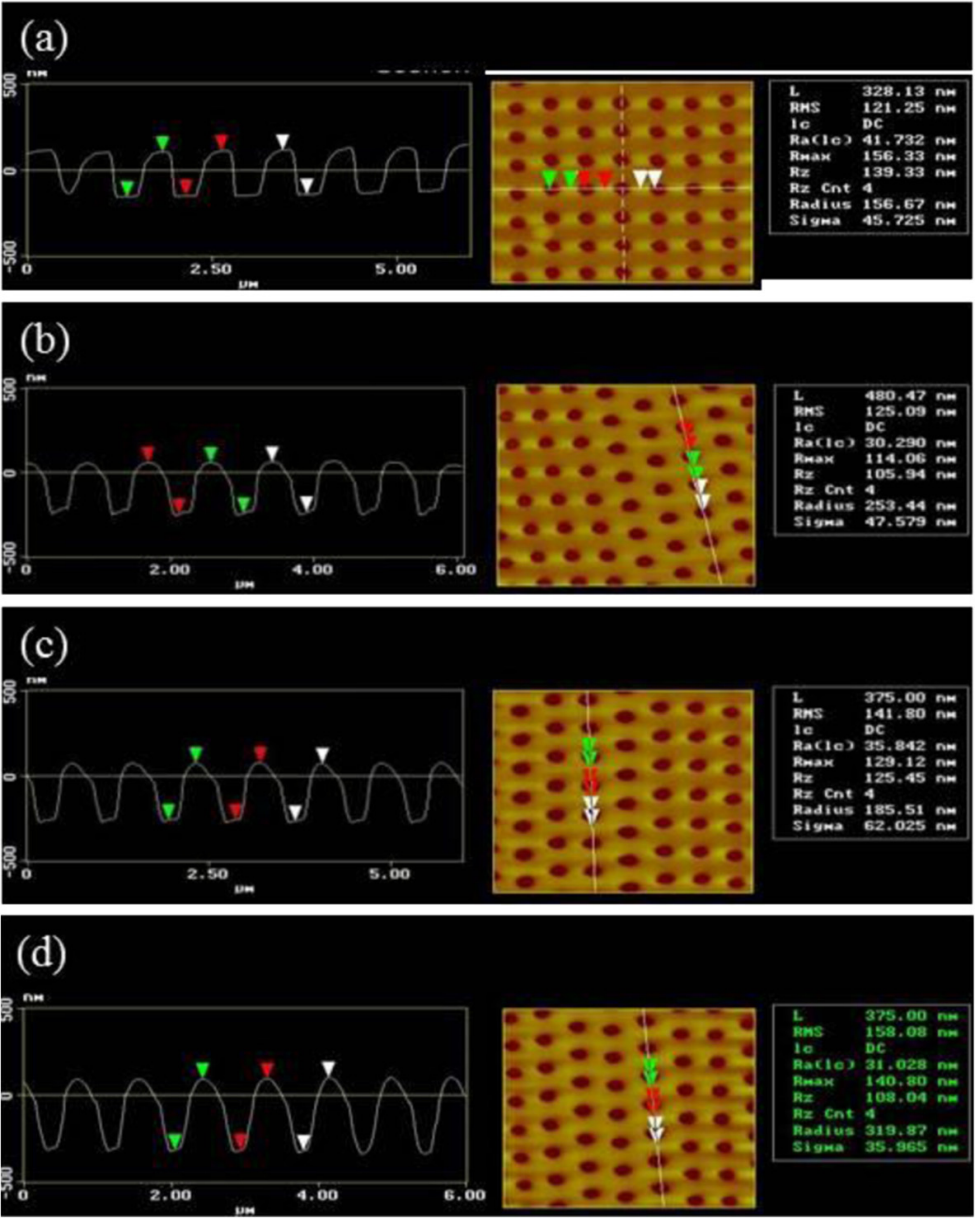
**AFM micrographs of measured NHA depths corresponds to different molding temperatures. (a)** 115°C, **(b)** 120°C, **(c)** 125°C, **(d)** 130°C.

It is noticeable and worth pointing out that the NHAs fabricated here have geometrically hemispherical bottom which can be potentially served as the backside reflector one-end open cavities for photon trapping. Next, a wide range of nanohole depths in the range of approximately 200 to 420 nm can be quickly and reliably replicated simply by changing the mold temperature as shown in AFM measurements of Figure 4a,b,c,d. It experimentally scanned five to seven areas for each sample from the center to the circumference and variation in fabricated NHAs in terms of replication depth, diameter and periodicity and was found to be negligible, showing a consistent replication over an area of 100-mm-diameter PC film. The section analysis and associated top views for various depths as a function of molding temperature reveals that the depth is linearly proportional to the molding temperature. Note that the injection nanomolding is widely controlled in the compact disk industry, which is technically proven to be a fast, large area with a high-throughput manufacturing process. The density of surface features can be readily tuned simply by changing another Ni stamp of different periodicity. The manufacture of Ni stamp adopts the commercially available electroforming process which is described elsewhere [30,31]. Generally, other anti-reflection nanotextured surfaces such as etching utilized anodization voltage to control the pitch over the surface feature density, while uniformity can be a serious issue over a large area. Notably, the depths of NHAs can be independently tuned by molding temperature in the present study.

**Figure 5:**
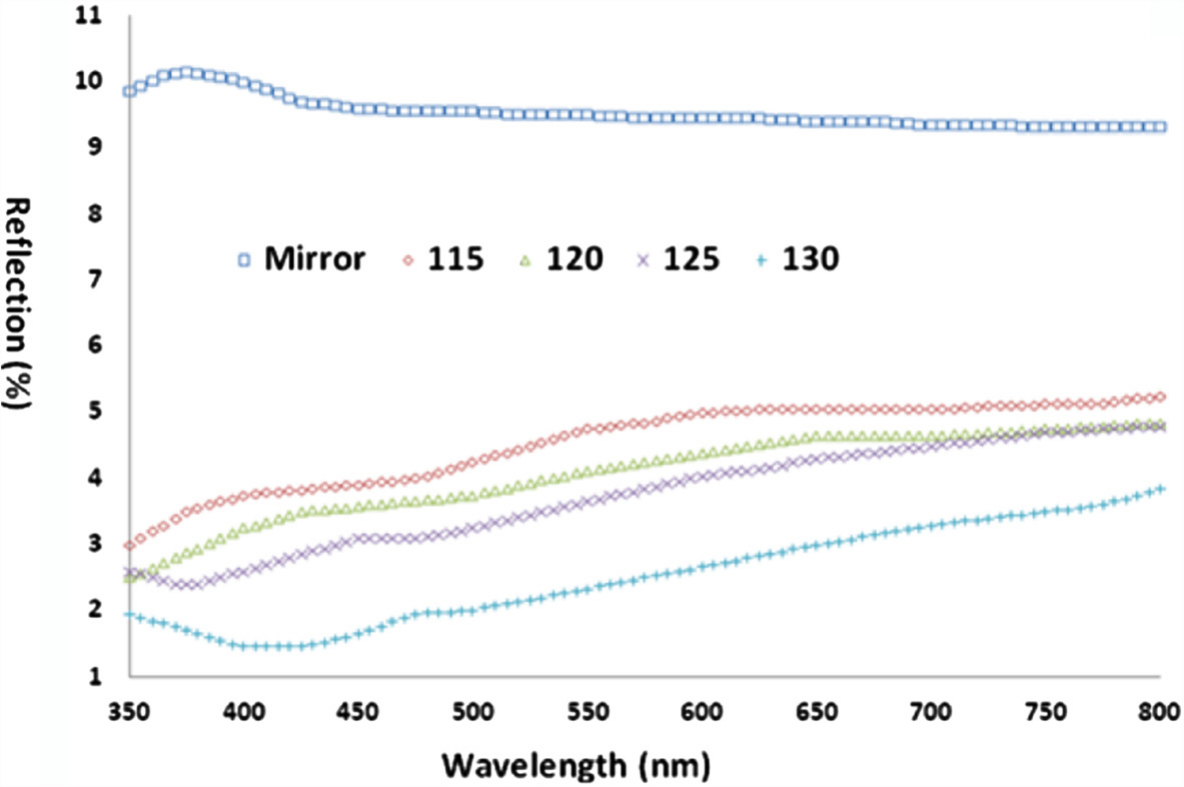
**Measured reflectivity of fabricated PC film and bare PC film.** Fabricated PC film with various depths of nanoinjected submicron holes and bare PC film as a function of the wavelengths. The mirror means the bare PC film, while the numbers of 115 to 130 corresponds to the molding temperatures in Celsius and associated depths can be referred to Figures 4 and 6, respectively.

**Figure 6:**
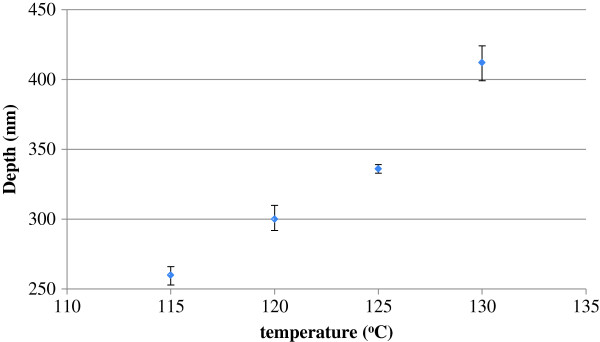
AFM measured depth of replicated nanohole arrays on PC film as a function of molding temperature.

Based on the above reliable replication of injection nanomolded textured PC film, we subsequently focus on the utility and potential practical applications as anti-reflection layers. Given the controlled geometry of the surface features with prescribed diameters, depth, and periodicity, textured PC film can be utilized as ideal nanoscale replication tools for template-assisted replication of nanostructured materials using nanoimprinting process. Furthermore, another important application of surface texturing is the enhancement and/or tunability of photon management. Bio-inspired structures include “moth eye” antireflective coatings and intentionally textured back contacts are two specific examples which have been shown as promising candidates to enhance the absorption and/or carrier collection efficiency of solar cells. In particular, large-area subwavelength surface texturing with tunable capability is highly desired. Measured reflection spectra of both bare and textured PC films are shown in Figure 5. Bare PC films can be considered as the mirror surface and exhibit a high average reflection of 9% to 10% over the explored wavelength range of 350 to 800 nm. The light reflection can be dramatically decreased to approximately 1.3% for the approximately 410-nm depth holes at the optical frequency of 420 nm. For other nanotextured surfaces with the same periodicity, the light reflection for different depths can be clearly discernible and approximately proportional to light reflection. The low reflectivity of nanotextured surfaces is vividly attributed to the bio-inspired NHA, without resorting to other methods such as tunability of refractive index typically utilized as light trapping in the deep trenches of the pores. The tendency for the reflection decrease due to the increase of NHA depth over the solar spectrum of 350 to 800 nm may be attributed to the smaller refractive index gradient with respect to structure depth [32]. Theoretically, the refractive index gradient plays a critical role in the significant suppression of broadband reflection through destructive interference such that the continuous change in refractive index causes the incident light to be reflected at different depths from the interface of air and anti-reflection coatings. Figure 6 shows the AFM measured depth of the replicated nanohole arrays on PC film as a function of the injection nanomolding temperature. It can be experimentally determined that molding temperature is an effective parameter to reliably control the depths of NHA over a large area.

**Figure 7:**
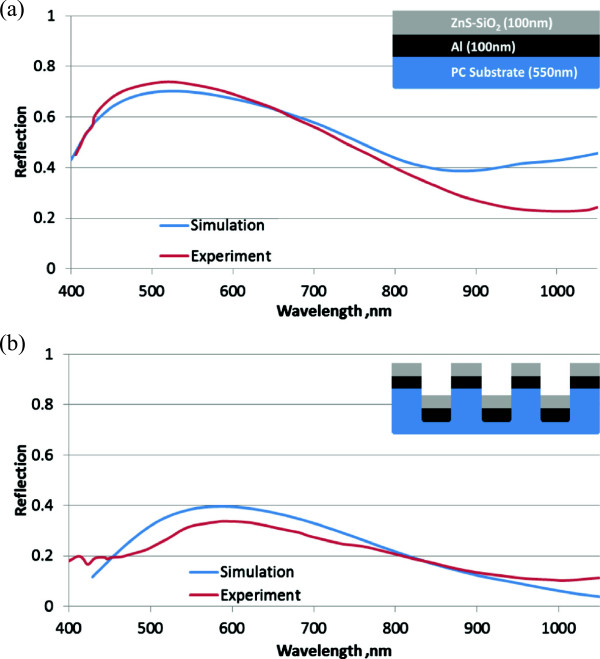
**Reflection spectra of mirror surface and nanohole array (NHA) structure with metallic and dielectric coating layers.** Simulated and experimentally measured reflection for **(a)** mirror surface and **(b)** NHA structure at normal incidence angle, respectively.

In the experimental implementation of the metallic and dielectric layers deposited on the PC substrate, the method of high-vacuum plasma-assisted deposition was used and both the metallic layer Al and dielectric layer ZnS-SiO_2_ films were deposited sequentially under the conditions of Class 100 cleanroom. The thickness of Al film is approximately 100 ± 20 nm and was measured by atomic force microscope with use of the kapton tape technique. Figure 7a shows reflection spectrum of the mirror surface, as well as the reflection spectrum of NHA with metallic and dielectric layers calculated with the use of the finite difference time domain (FDTD) approach. The increased reflection was measured due to extra coating layers of Al (100 nm) and ZnS-SiO_2_ (100 nm), resulting in the highest reflection at 520 nm and reflection value of almost 0.73 for the mirror surface. It is observed that a similar trend can be obtained from the FDTD analysis. Again, the reflection from the similar coating layers on the NHA is significantly lower as compared to the mirror surface, which corresponds to the highest reflection at 589 nm and reflection value of almost 0.33 as shown in Figure 7b. Despite the similar coating layers on the same PC substrate and the same refractive index, NHA configuration does exhibit one important feature of shifted peak of reflection and can potentially function as an ultrasensitive sensing device.

## Conclusions

In summary, a versatile and rapid process is presented based on the well-established injection nanomolding of PC polymer for the controlled nanotexturing of NHA surfaces over large areas with tunable depth topography. In addition, with the change of master Ni stamp, feature size diameter and density/periodicity can also be adjusted accordingly. The NHA-engineered surfaces exhibit a functional optical property that can be optimized for anti-reflection coatings. The proposed technology of rapidly replicated NHA surfaces may be used for efficient and cost-effective solar cells, highly light extracted light-emitting diodes (LED) and self-cleaning surfaces. The scalability of the process can be sufficiently addressed due to the reduced cycle time of 4 s and is fully compatible with the well-established mass production of DVD/BD industries. This work presents an important advance in the rapidly growing field of nanomanufacturing. Furthermore, we have also experimentally demonstrated an approach to quantitatively control transmission of light through NHA and multilayer coating of both dielectric and metallic layers with the potential use of sensing applications. The future work can be extended to the transmission of light through current NHA/multilayer structures and geometry-dependent selectivity in terms of both frequency and resonant width.

## Competing interests

The authors declare that they have no competing interests.

## Authors’ contributions

YKF designed the experiments, analyzed the data, and wrote the paper. CCP performed the experiments and measurements. CTH performed the simulations, helped with the revisions of the manuscript and preparation of response letters. All authors discussed the results, commented on, and approved the final manuscript.
